# Simultaneous bilateral hip replacement reveals superior outcome and fewer complications than two-stage procedures: a prospective study including 1819 patients and 5801 follow-ups from a total joint replacement registry

**DOI:** 10.1186/1471-2474-11-245

**Published:** 2010-10-25

**Authors:** Emin Aghayev, Andreas Beck, Lukas P Staub, Daniel Dietrich, Markus Melloh, Weniamin Orljanski, Christoph Röder

**Affiliations:** 1 Institute for Evaluative Research in Orthopedic Surgery, University of Bern, Stauffacherstrasse 78, 3014 Bern, Switzerland; 2 Department of Orthopedic Surgery, Spital Langenthal, St. Urbanstrasse 67, 4901 Langenthal, Switzerland; 3 Institute for Mathematical Statistics and Actuarial Science, University of Bern, Sidlerstrasse 5, 3012 Bern, Switzerland; 4 Department of Orthopedic Surgery, University of Otago, Private Bag 1921, Dunedin, New Zealand; 5 Department of Orthopedic Surgery, Vienna Private Clinic, Pelikangasse 15, 1090 Vienna, Austria; 6 Department of Orthopedic Surgery, University of Bern, Freiburgstrasse 18, 3010 Bern, Switzerland

## Abstract

**Background:**

Total joint replacements represent a considerable part of day-to-day orthopaedic routine and a substantial proportion of patients undergoing unilateral total hip arthroplasty require a contralateral treatment after the first operation. This report compares complications and functional outcome of simultaneous versus early and delayed two-stage bilateral THA over a five-year follow-up period.

**Methods:**

The study is a post hoc analysis of prospectively collected data in the framework of the European IDES hip registry. The database query resulted in 1819 patients with 5801 follow-ups treated with bilateral THA between 1965 and 2002. According to the timing of the two operations the sample was divided into three groups: I) 247 patients with simultaneous bilateral THA, II) 737 patients with two-stage bilateral THA within six months, III) 835 patients with two-stage bilateral THA between six months and five years.

**Results:**

Whereas postoperative hip pain and flexion did not differ between the groups, the best walking capacity was observed in group I and the worst in group III. The rate of intraoperative complications in the first group was comparable to that of the second. The frequency of postoperative local and systemic complication in group I was the lowest of the three groups. The highest rate of complications was observed in group III.

**Conclusions:**

From the point of view of possible intra- and postoperative complications, one-stage bilateral THA is equally safe or safer than two-stage interventions. Additionally, from an outcome perspective the one-stage procedure can be considered to be advantageous.

## Background

Total joint replacements represent a large part of day-to-day orthopaedic routine. For the aging population, total hip arthroplasty (THA) has become a key treatment for re-establishing independence and quality of life. A substantial proportion of patients undergoing unilateral total hip arthroplasty require a contralateral treatment thereafter [1-3]. Since Ritter and Randolph (1976) performed the first detailed study of the functional outcome of simultaneous bilateral THA, there has been an ongoing discussion regarding benefits and disadvantages of one-stage versus two-stage procedures [4]. A number of articles report advantages of simultaneous operations, or at least similar results of one-stage versus two-stage procedures [1,2,5-12]. The largest sample described consisted of 461 patients with a mean follow-up time of 3 years, although functional outcome was not assessed [10].

In 1965 M.E. Müller started a systematic collection of THA outcome data and developed a documentation system that culminated in IDES, the International Documentation and Evaluation System for total hip and knee arthroplasty. IDES and precursors have collected prospective information about 48,000 primary THA, 12,000 revision THA, and 77,000 follow-ups from 65 hospitals in Europe. The richness of information in the database makes possible comparison of different timings of bilateral THA.

In the current study we compared simultaneous bilateral THA (Group I) with early (within six months) (Group II) and delayed (within five years) two-stage bilateral THA (Group III) regarding complications and functional outcome over a five-year follow-up period. The two surgeries in the delayed two-stage group were separately analyzed. A large cohort of Charnley class A patients with unilateral THA were used as reference group for graphical comparisons.

## Methods

Our study is based on the IDES (International Documentation and Evaluation System) hip registry of the Institute for Evaluative Research in Orthopaedic Surgery at the University of Bern. The history and administration of the registry have been previously described [13,14]. Table 1 shows the variables used for the analysis.

**Table 1:** International Documentation and Evaluation System variables used for the study.

Variable	Answer options
Date of birth	

Date of surgery	

Gender	male
	female

Operation side	right
	left

Diagnosis	osteoarthritis
	developmental displasia
	inflammatory arthritis
	fracture
	miscellaneous

Pain	none
	mild
	moderate
	severe
	intolerable

Walking capacity restriction	>60 min
	31 min to 60 min
	10 min to 30 min
	<10 min
	impossible

Flexion	>90°
	71° to 90°
	30° to 70°
	<30°
	stiff

Harris Hip Score	

Intraoperative complications	none
	perforation
	proximal fracture
	distal fracture
	fracture of trochanter
	tendency to dislocate
	vascular
	pelvic perforation
	other

Systemic postoperative complication	none
	deep thrombosis
	pulmonary embolism
	cardiovascular
	respiratory
	gastrointestinal
	urological
	CNS
	other

Local postoperative complication	none
	hematoma
	dislocation
	neuropraxia
	wound dehiscence
	superficial
	deep infection
	sinus
	other

Follow-up date	

### Sample characteristics

Institutional review board approval at our center was not required as it utilized existing anonymous observational data. The information was derived from 41 hospitals in 8 European countries (Switzerland, France, Germany, Italy, The Netherlands, Spain, Belgium, and Austria) where standardized datasets have been collected in a prospective, systematic and consecutive mode. All cases with bilateral THA operated between 1965 and 2002 and at least one follow-up per hip within the first five postoperative years were selected. If there were two or more follow-ups per year, the one closest to the middle of the year was selected. No case with revision was included. Patients with Charnley classes A (except for graphical comparisons) and C were excluded since the first group has unilateral hip disease by definition and the latter group has comorbid conditions influencing the functional outcome [15]. Patients under 19 years of age were also excluded, but no other exclusions based upon the primary diagnosis were made. The database query resulted in 1819 patients with 5801 follow-ups (3.2 follow-ups per patient) within the first five postoperative years.

According to the timing of the two operations the patient sample was divided into three groups. The first group of 247 patients had undergone simultaneous bilateral THA. The second group of 737 patients had early two-stage bilateral THA, i.e. the second operation was performed within six months after the first. The third group of 835 patients had delayed two-stage bilateral THA with an interval of six months to five years.

In addition to the 3 groups with bilateral hip disease, a 4^th ^reference group including patients with unilateral hip disease and no other comorbid condition affecting mobility and motion (Charnley class A) was created [15]. This reference group was comprised of 8402 patients and solely used for graphical comparisons. The graphical reference of this group wants to provide the reader with a visual comparison between the functional outcome of the three groups under study and the classical reference group of THA patients with an isolated and unilateral hip disease and no other condition affecting mobility and motion.

Outcome variables were pain, walking capacity, flexion, Harris Hip Score (HHS), a composite score summarizing items of pain, mobility, motion and some activities of daily living into a sum score from 0-100, [16] as well as intraoperative, systemic and local complications. Certain categories of pain and function from the IDES database were combined for our study. Pain was classified as none/mild, moderate, or severe/intolerable; walking capacity was classified as more than 60 minutes, 31-60 minutes, 10-30 minutes, or less than 10 minutes/not possible; the range of hip flexion was classified as >90°, 71°-90°, 30°-70°, or <30°/stiff. We defined a desired outcome as no or mild hip pain, a walking capacity of longer than 60 minutes, and a range of hip flexion of >90°. We hypothesized that the outcomes of the three groups with bilateral surgeries are affected by the timing of the second surgery in relation to the first one.

### Statistical analysis

Chi-square test was used for comparing proportions between the study groups. All follow-up examinations were grouped on the basis of annual follow-up intervals. Multivariate modeling (generalized estimating equations method, GEE) was applied for globally assessing group differences with regard to outcome variables over time. Correction factors of gender, age, diagnosis, and baseline values were employed for the respective outcome variables. Additionally, for each outcome variable and each follow-up year the Cochran-Mantel-Haenszel test was used to compare the patient groups. Stratification factors were the same as for the multivariate model. Bonferroni-Holm adjustments for each outcome variable were set to account for multiple testing over the five follow-up years. The empirical proportions of desired outcomes and related 95% percent confidence intervals were plotted to graphically display the variability within the groups. The level of significance was set to 0.05 throughout the study. All statistical analyses were conducted using SAS 9.1 (SAS Institute Inc, Cary, NC).

### Literature review

A systematic search of the literature was conducted to identify studies comparing simultaneous versus two-stage bilateral THA. MEDLINE was searched with the following free text and MeSH search terms: hip arthroplasty, hip disease, bilateral THA, simultaneous OR one stage OR two stage THA. Additionally, the reference list of each eligible article was screened for other relevant publications (cross reference search) to identify additional studies. The articles were included if the aim of the study was to compare one versus two stage bilateral THA. There was no language, age or publication year restriction.

## Results

The demographic characteristics of the study groups are shown in table 2.

**Table 2:** Demographic characteristics of the studied groups.

	Number of patients	Age		Proportion of women	Treated between (ys)	80% of treatments performed between (ys)
					
		Range	Mean			
Group I	247	22-85	59	53%	1978-2001	1989-1999

Group II	737	20-88	62	50%	1968-2002	1984-1999

Group III	835	22-87	63	49%	1980-1998	1984-1994

Reference group (unilateral)	8402	20-94	65	51%	1982-2000	1985-1998

### Comparison of groups - preoperative status

Table 3 shows the distribution of diagnoses in the groups. Table 4 shows in bold the percentage of patients with severe or intolerable pain, walking capacity below 10 minutes, and hip flexion range below 30 degrees. The simultaneous group I had the lowest proportion of patients with severe/intolerable pain, and group II had the highest percentage of painful hips. The simultaneous group I had the least compromised preoperative ambulation; the second and third group were similar and considerably worse. Regarding range of motion the group III was the best and the worst was group II. Global inter-group differences were significant for hip pain, walking capacity and flexion (p < 0.001 for all variables).

**Table 3:** Distribution of diagnoses. Global inter-group difference was significant (chi-square test: p < 0.001).

	Group I: 247		Group II: 737		Group III: 835		Group IV: 8402	
**Diagnosis**	**n**	**%**	**n**	**%**	**n**	**%**	**n**	**%**

Osteoarthritis	173	**70.0**	532	**72.2**	663	**79.4**	6391	**76.1**
Dysplasy	43	**17.4**	99	**13.4**	85	**10.2**	665	**7.9**
Inflammation	15	**6.1**	40	**5.4**	25	**3.0**	103	**1.2**
Fracture	1	**0.4**	9	**1.2**	6	**0.7**	689	**8.2**
Miscellaneous	15	**6.1**	57	**7.7**	56	**6.7**	554	**6.6**

**Table 4:** Preoperative pain, walking capacity and flexion.

	Group I: 247	Group II: 737	Group III: 835
**Pain**			

none/mild	6%	4%	2%
moderate	34%	26%	31%
severe/intolerable	**60%**	**70%**	**67%**

**Walking capacity**			

>60 min	11%	6%	5%
31 min to 60 min	18%	11%	14%
10 min to 30 min	26%	18%	17%
<10 min/impossible	**45%**	**65%**	**64%**

**Flexion**			

>90°	19%	9%	14%
71° to 90°	39%	42%	48%
30° to 70°	31%	36%	31%
<30°/stiff	**11%**	**13%**	**7%**

Global inter-group differences were significant for hip pain, walking capacity and flexion (chi-square test: p < 0.001 for all three variables).

### Comparison of groups - outcome

**Figure 1:**
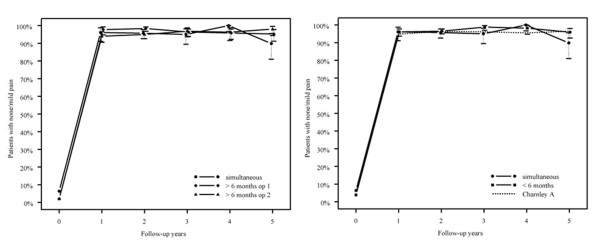
**Proportion of patients with none/mild pain in the groups**.

**Figure 2:**
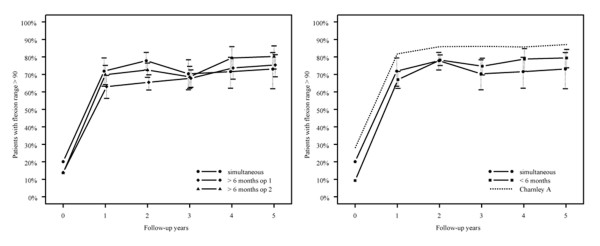
**Proportion of patients with flexion range >90°**.

**Figure 3:**
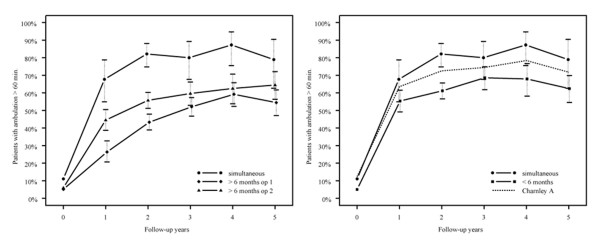
**Proportion of patients with ambulation >60 min**.

The excellent outcome regarding hip pain, walking endurance and flexion is shown in figures 1, 2 and 3 where the empirical frequencies (uncorrected for gender, age, diagnosis, and baseline values for respective outcome variables) are displayed. The reference group is displayed as dotted line (figures. 1, 2, 3). Whereas hip pain did not differ between the groups (p = 0.1, figure 1), the best walking capacity was observed in the simultaneous group I and the worst in group III after the first surgery (p < 0.001, figure 3). Compared with the simultaneous group I, the odds for excellent walking capacities in group II were 0.49, 0.25 in group III after the first and 0.36 after the second surgery (p < 0.001 for all comparisons).

According to the GEE method there was a significant difference in range of postoperative hip flexion between at least two groups. The individual group comparisons did then show a significant difference between group III after the first surgery and group I (p < 0.001, figure 2). The odds for excellent flexion in group III after the first surgery were 0.63 compared with group I.

Similarly, group I had the best mean HHS (94.2, range 37-100), followed by group II (92.6, range 51-100) and group III after both surgeries (89.9, range 48-100). This difference in HHS was significant between groups I and III (p < 0.001), and II and III (p = 0.040).

A separate intragroup comparison between the first and second surgery in group III showed significant difference regarding walking capacity (p < 0.001) and hip flexion (p = 0.009). The odds for excellent walking capacity were 0.68 after the first surgery compared to the second one. For excellent flexion they were 0.77 after the first surgery.

### Comparison of groups - complications

The simultaneous group I showed an intraoperative complication rate of 3.2% during the first operation and of 6.9% during the second one (table 5). Fracture of the trochanter was the most frequent complication during both the first (1.6%) and the second operation (4.0%). The trochanter fracture rate, an important complication for functional outcome, was the highest in group I (5.7% in group I versus 3.5% in group II and 4.4% in group III after both operations) but not significantly different to the other groups neither in combined (OP1 + OP2) nor in separated comparisons.

**Table 5:** Rate of complictions.

		**OP1**			**OP2**		**OP1 + OP2**		
		
**IntaOP complications**	**Group I**	**Group II**	**Group III**	**Group I**	**Group II**	**Group III**	**Group I**	**Group II**	**Group III**
		
shaft perforation	-	1	2	1	-	2	1	1	4
proximal Fx	2	4	9	-	2	9	2	6	18
Fx of trochanter	4	13	16	10	13	21	14	26	37
tendency for luxation	-	7	9	4	10	5	4	17	14
vascular injury	-	-	2	-	-	1	-	-	3
pelvic perforation	-	9	6	1	4	4	1	13	10
other	2	6	3	1	2	6	3	8	9
		
***total %***	***3.2***	***5.4***	***5.5***	***6.9***	***4.2***	***5.6***	***10.1***	***9.6***	***11.1***
		
**Systemic complications**	**Group I**	**Group II**	**Group III**	**Group I**	**Group II**	**Group III**	**Group I**	**Group II**	**Group III**
		
deep thrombosis	-	6	16		1	5	-	7	21
pulmonary emboly	1	6	6		4	3	1	10	9
cardiovascular	3	5	14		6	17	3	11	31
respiratory	-	7	8		1	3	-	8	11
gastrointestinal	3	13	13		8	9	3	21	22
urological	11	12	25		14	16	11	26	41
CNS	-	3	5		3	5	-	6	10
other	8	6	5		7	8	8	13	13
		
***total %***	***10.5***	***7.9***	***10.8***		***6.0***	***7.7***	***10.5***	***13.8***	***18.5***
		
**Local complications**	**Group I**	**Group II**	**Group III**	**Group I**	**Group II**	**Group III**	**Group I**	**Group II**	**Group III**
		
haematoma	11	38	44	12	34	49	23	72	93
dislocation	1	2	4	3	5	5	4	7	9
neuropraxia	-	2	7	1	4	7	1	6	14
wound dehiscence	-	5	2	-	1	5	-	6	7
		
***total %***	***4.9***	***6.4***	***6.7***	***6.5***	***6.0***	***7.7***	***11.4***	***12.4***	***14.4***

With 10.5% the rate of postoperative systemic complications was the lowest of all groups (table 5). The most common complication was a urinary tract infection during hospital course (4.5%).

Similarly, group I also had the lowest rate of postoperative local complications (11.4% after both operations). A hematoma was the predominant local complication during both the first (4.5%) and the second operation (4.9%).

In group II the rate of intraoperative complications was higher than in group I during the first (5.4%) but lower during the second (4.2%) intervention (table 5). The most common complication also was a trochanter fracture (1.8% for both the first and the second intervention).

The 13.9% of postoperative systemic complications were also higher than group I (table 5). The predominant complications were a urinary tract infection (1.6% after the first and 1.9% after the second operation) and postoperative obstipation (1.8% after the first and 1.1% after the second operation).

The sum of postoperative local complications after both operations was 12.4% (table 5). The predominant local complication was a hematoma during both the first (5.2%) and the second operation (4.6%).

Group III presented the highest total complication rates. There were 5.5% of intraoperative complications during the first and 5.6% during the second operation, while postoperative local complications were 6.7% after the first and 7.7% after the second intervention. Systemic complication rates were 10.8% after the first and 7.7% after the second operation (table 5). The most common intraoperative complication was fracture of the trochanter (1.9% during the first and 2.5% during the second surgery). As in the other groups a hematoma was the most frequent postoperative local complication (5.3% after the first and 5.9% after the second surgery). The most common postoperative systemic complications were deep vein thrombosis, cardiovascular and urological complications summing up to 6.2% after the first and 4.6% after the second operation.

## Discussion

We compared simultaneous bilateral THA with early and delayed two-stage bilateral THA regarding complications and functional outcome over a five-year follow-up period.

Lindberg and Sjöstrand (1972) estimated that approximately one-third of patients with primary osteoarthritis of the hips would need bilateral surgery [17]. This suggests the considerable importance of the comparison of one-stage versus two-stage bilateral THA.

In our study, a large number of European patients who had undergone bilateral total hip arthroplasty were analyzed in terms of three variables: walking endurance, hip pain and hip flexion range. Additionally, the rates of intraoperative and postoperative local and systemic complications and the Harris hip score were assessed.

Despite the differences regarding preoperative functional status, postoperative pain alleviation did not differ between the three groups during the first 5 follow-up years. Pain relief after THA is immediate, constant, and long-lasting, and it is independent of the preoperative pain level and demographic factors [18,19].

In contrast, the proportion of patients walking longer than 60 minutes was 20% higher in the simultaneous group than in group II, and 28% higher than in group III at the 5 year follow-up. A possible explanation for this difference is the younger average age of group I which was 59 years, compared with 62 years in the second and with 63 years in the third groups. Moreover, unlike preoperative pain, preoperative walking capacity influences postoperative outcome and the preoperative walking capacity of group I was far better than that of the other groups [18]. Nevertheless, even after correction for preoperative condition and age in the statistical model the differences in walking capacity remained significant. In addition, the fact that patients with a simultaneous arthroplasty on both sides undergo only one rehabilitation and mobilization program seems advantageous. A single hospital stay is required for patients to learn to walk with altered proprioception, improved flexion range and pain alleviation. In contrast, bilateral hip disease with solely unilateral THA may also show clear improvement in postoperative walking endurance-but not the optimal one, since the contralateral untreated side remains painful and can limit overall function. Wykman and Olson stated that in bilateral hip disease, optimal function is not entirely regained until both hips have been replaced [20]. Optimal function can be achieved more quickly with a one-stage than the two-stage procedure [20]. Ritter et al. reported that in the contralateral osteoarthritic hip of a patient with one THA, the patient has a 78.5% chance of progression of his osteoarthritis and a 54% chance of requiring a second THA within 10 years [3]. Such a progression of contralateral osteoarthritis should be anticipated in patients with bilateral hip disease, which suggests treatment with simultaneous THA if the patient's overall health condition is permissive.

In group II, a second operation and yet another rehabilitation were undertaken after the first operation and the patient's habituation to the new prosthesis. In elderly people who make up a major part of the patients undergoing bilateral THA and who have a more limited habituation potential, a two-stage operation may result in suboptimal improvement of walking endurance. Weinstein et al. described one-stage bilateral THA as a safe and effective option even for patients over age 75 [21].

The comparison of the patients` status after the first and second operation in group III showed significantly different walking capacity and flexion in favor of the second intervention. Thus, in the time between the first and second operation, the patients with bilateral hip disease did not benefit from the full potential outcome of their therapy and though they were older after the second surgery their function improved beyond the status after the first THA. This is one of the main findings in favor of a consequent one-staged or early two-staged bilateral hip replacement intervention.

The possibly increased likelihood for intra- and postoperative local and systemic complications is the most frequently cited argument against simultaneous bilateral THA. In our study, however the analysis of complication rates showed an advantageous situation in group I with the simultaneously operated patients. The rate of intraoperative complications was comparable to that of group II; however, the frequency of postoperative local and systemic complications in group I was the lowest of all the three groups. The higher rate for trochanter fractures in group I during the second operation was not significantly different to that in the other groups. It did also not considerably influence the average postoperative pain, flexion and walking capacity after the simultaneous procedure. This may be explained by the fact that trochanteric fractures are mostly stable and do not require an additional treatment [22].

The worst group regarding complications was group III. The most prevalent complications were postoperative ones, including deep vein thrombosis and cardiovascular as well as urological events. These differences were observed despite the similar demographic characteristics of the groups. Parvizi also found that patients treated with two-stage bilateral THA arthroplasty had more complications, most commonly anemia and wound drainage [8].

In early publications comparing bilateral with unilateral THA, a higher incidence of pulmonary embolism and somewhat increased morbidity for bilateral THA was reported [4,23,24]. However, as concluded by Ritter and Stringer, the introduction of hypotensive anesthesia, improvement of operative environment, anticoagulation therapies, and early postoperative ambulation of patients have lead to a decrease in complication rates after bilateral THA [23]. In a detailed prospective study, Cammisa studied 35 adults with different preoperative status including myocardial infarction and pharmacologically controlled hypotension. The authors reported equal safety for both one-stage bilateral and unilateral THA without pulmonary emboli, myocardial infarctions, or other similar complications in the perioperative period [25]. Similarly, Salvati et al. found no differences in postoperative and long-term complications between one- and two-stage bilateral THA [10].

Literature concerning comparison of simultaneous and two-stage bilateral THA is summarized and compared in table 6. The 10 articles were published between 1978 and 2007 and they cover patients treated between 1970 and 2006. The studies included between 30 and 461 patients and they show predominantly similar functional outcomes for simultaneous and two-stage procedures with a maximum average intraoperative interval of 1.82 years. Only Berend reporting on cementless bilateral THA documented a significantly increased need for postoperative inpatient rehabilitation services, as well as the fact that significantly fewer patients in the simultaneous group had met physical therapy goals before discharge to home [5]. In contrast, according to Schiessel patients prefer the simultaneous procedure because they undergo the process of operation, mobilization, and rehabilitation only once [11]. A better functional outcome after one-stage procedures is also reported for very stiff hips with a preoperative range of motion below 50° [2].

**Table 6:** The found literature on comparison between one-stage and two-stage bilateral THA.

N	Authors	Year	Period	Study	**Number of pat**.	Inter-operative days	FU (months)	Functional outcome	Complications	Pro/contra
1	Alfaro-Adriàn	1999	1989-95	1/2 stage	202(95/107)	0/60-730(300)	0	similar	>blood transfusions	pro: <hospital stay, costs

2	Berend KR	2007	1997-05	1/2 stage	277(167/110)	0/14-730(240)	30(6-108)	early: <function	>blood transfusions >revisions	contra: <reimbursement

3	Bhan	2006	1996-01	1/2 stage	168(83/85)	0/90-210	60(48-96)	similar	>blood transfusions	pro: < hospital stay

4	Eggli	1996	1982-92	1/2 stage	255(64/63/128)	0/>42/42-180	>18	similar; >stiff hip	similar	pro: <hospital stay, costs

5	McBryde	2007	1994-06	1/2 stage	92(37/55)	0/1-365	15(1-60)/34(1-131)	early better	similar, >intubation time	pro: <hospital stay, costs

6	Parvizi	2006	1997-04	1/2 stage	196(98/98)	0/25-303(138)	0	similar	<blood transfusions <complications	pro: <costs, >rehabilitation

7	Reuben	1998	1991-94	1/2 stage/unilat	154(7/8/139)	0/7/unilat	0	-	-	pro: < costs

8	Salvati	1978	1970-76	1/2 stage	461(122/134/205)	0/same hosp/2nd hosp	36(12-96)	similar	similar	pro: <hospital stay, costs, OP time

9	Schiessel	2005	1996-02	1/2 stage	30(15/15)	0/120-665(485)	66(SD 19.5)	similar (subjectively>)	similar	pro: <hospital stay, costs

10	Shih	1985	1979-82	1/2 stage	35(20/15)	0/14-365	12/17.7	similar but <ROM	similar	pro: <hospital stay, OP time

The column "Study" shows the studied subgroups. The column "Number of patients" represents the total number of studied patients and number of subgroups in parentheses. The inter-operative interval for the subgroups is shown in the column "Inter-operative days" with mean values for the two-stage subgroup included in parentheses, if available. In the column "FU (month)" the mean follow-up time is given with the range of follow-up time or standard deviation (SD) in parentheses, if available. Note that ">" and "<" mean "more/better" respectively "less/worse" in the simultaneous subgroup.

The above mentioned articles report similar complication rates. However, Berend documented a significantly higher re-operation rate, more inpatient complications and adverse events in patients undergoing simultaneous bilateral THA in the lateral decubitus position although the author does not list them [5]. On the other hand, Parvizi reported fewer complications in the simultaneous group [8]. The remaining literature describes no significant differences in complication rates between simultaneous and two-stage bilateral THA.

Three out of 10 articles noticed a higher need for blood transfusion in the simultaneous group, whereas the article by Parvizi described a lower need [1,5,6,8]. McBryde noticed significantly longer anesthetic time in the simultaneous group than in two-stage procedure if compared to the time of each single operation. He accounted for this observation with the time needed to undrape, reposition the patient, redrape and prepare the patient again.

Out of 10 articles, 9 were in favor of simultaneous bilateral THA due to better cost efficiency, shorter hospital stay (n = 9) and shorter operation times (n = 2). Only Berend was against simultaneous bilateral THA [5]. His additional major argument was that of a lower potential reimbursement for hospital and surgeon [5].

Weinstein compared simultaneous bilateral THA in patients older and younger than 75 years [21]. Although, as expected, the older group owed more complications, the author documented excellent functional outcomes in both groups and concluded by favoring simultaneous bilateral THA, even for patients older than 75 years [21].

In the first group we had more patients with arthritis secondary to developmental dysplasia, which usually occurs earlier than primary coxarthritis, affecting mostly younger and more active individuals [11]. Nevertheless, the overall age distribution between the groups showed an average age difference of no more than three years. Concerning bilateral THA for dysplastic coxarthritis it is known that despite greater efforts in rehabilitation, patients prefer the simultaneous bilateral implantation since they need to undergo the process of operation, mobilization, and physiotherapy only once [11].

Our study has limitations that need to be considered when interpreting the results. We studied main outcome parameters such as walking endurance, pain, flexion, and complication rates. Obviously there are other outcome variables that depend on the timing of the two interventions. Further studies with the assessment of these variables are necessary for a more comprehensive comparison of the different possible operative intervals in bilateral THA. Furthermore, the study represents a retrospective analysis of prospectively, systematically and consecutively collected data. Despite this setup, the multitude of centers included and the large time frame carry with them the potential for selection bias in such a non-monitored data collection endeavor. If there is selection bias, however, it should rather be a non-systematic one since none of the hospitals was aware of the goal of the current study and could have selectively included or excluded cases to their advantage. Therefore observed effects are rather diminished than amplified. Also, the simple fact of lack of detailed comorbidity reporting in the registry and hence impossibility to adjust for them in the statistical model may be responsible for higher prevalence for systemic complications in group III, since it could have been reasonable for patients under certain clinical conditions to undergo delayed two-stage procedures. We can therefore not clearly decide if comorbidity status or two the separate surgeries with anesthesia are responsible for this phenomenon.

It can be stereotypically concluded that a randomized controlled trial is the best option to study the effects of bilateral THA timing but given the restrictions of feasibility, cost, ethics, case numbers and follow-up time, a prospective cohort study, especially in the framework of a registry, may still be the best trade-off between invested efforts and resources and evidence level that the findings can generate.

## Conclusions

Summarizing our results, from the point of view of possible intra- and postoperative complications, in patients with bilateral hip disease and adequate medical condition simultaneous bilateral total hip arthroplasty must be seen as equally safe or even safer than two-stage interventions. From an outcome perspective, the one-stage procedures can be advantageous.

## List of abbreviations used

THA: Total Hip Arthroplasty; IDES: International Documentation and Evaluation System; HSS: Harris Hip Score; GEE: generalized estimating equations method; OP: operation; UNILAT: unilateral; ROM: range of motion.

## Competing interests

The authors declare that they have no competing interests.

## Authors' contributions

EA is the principal investigator. He performed the study including statistical analysis and drafted the manuscript. AB performed literature review and helped in drafting manuscript. LS and DD participated in the statistical analysis. MM and WO supervised the study and drafting the manuscript. CR conceived the study and participated in coordination and supervision of the study. All authors participated in the study design as well as read and approved the final manuscript.

## Pre-publication history

The pre-publication history for this paper can be accessed here:

http://www.biomedcentral.com/1471-2474/11/245/prepub
